# Iris pigment epithelial cysts in acute lymphoblastic leukemia-a case report

**DOI:** 10.1186/s12886-025-04419-8

**Published:** 2025-10-17

**Authors:** Kar Yong Chong, Yong Zheng Wai, Nurhayati Abdul Kadir, Lik Thai Lim, Raja Muhamad Zul Hatta, Wai Seng Chiang

**Affiliations:** 1 Ophthalmology Department, Duchess of Kent Hospital, Sandakan, Malaysia; 2 Universiti Malaysia Sarawak (UNIMAS), Sarawak, Malaysia; 3 Pathology Department, Queen Elizabeth Hospital, Kota Kinabalu, Sabah Malaysia

**Keywords:** Iris pigment epithelial cysts, Acute lymphoblastic leukemia, Bone marrow aspiration and trephine biopsy, Bone marrow immunophenotyping

## Abstract

**Background:**

Iris cysts are classified as either primary or secondary, with further subcategorization based on the tissue of origin. The most common type is the primary iris pigment epithelial (IPE) cyst. We report a rare case of bilateral IPE cysts in a patient with acute lymphoblastic leukemia (ALL).

**Case presentation:**

A 3-year-old male toddler initially presented with signs and symptoms of anemia. Multiple lymph nodes were palpable throughout the body, and hepatosplenomegaly was noted. An incidental finding of cystic lesions at the margins of both pupils prompted referral to the ophthalmology team. There was no family history of malignancy or similar eye conditions. Ophthalmic examination revealed IPE cysts in both eyes (BE). Fundus examination showed a dull macula, and tortuous vessels, with no retinal hemorrhages observed. Intraocular pressure was normal. Petechial rashes were present on both eyelids.

A full blood count revealed pancytopenia, and a peripheral blood film (PBF) showed a leucoerythroblastic picture without obvious blast cells. Bone marrow aspiration and trephine biopsy (BMAT) were performed. Trephine biopsy and bone marrow immunophenotyping were suggestive of B-cell acute lymphoblastic leukemia (B-cell ALL) with aberrant CD58 expression. Cytogenetic analysis revealed hyperdiploidy, a favourable prognostic marker. The patient was started on chemotherapy. Following initiation of chemotherapy, the IPE cysts decreased in size.

**Conclusion:**

In this case, the IPE cysts were likely associated with ALL. This rare occurrence may raise awareness of a potential link between IPE cysts and hematological malignancies, and could pave the way for future research to elucidate the underlying pathogenesis and treatment implications.

**Supplementary Information:**

The online version contains supplementary material available at 10.1186/s12886-025-04419-8.

## Background

Iris cysts have been reported in various clinical contexts. A classification system for iris cysts was proposed by Shields in 1981, categorizing them as either primary or secondary, with further subcategorization based on the tissue of origin (Table 1). [1, 2] The most common type is the primary iris pigment epithelial (IPE) cyst. [2] Most IPE cysts are asymptomatic and rarely progress or cause visual complications. [1] They are often discovered incidentally during routine slit-lamp or microscopic eye examinations. [4] Imaging techniques such as ultrasound biomicroscopy (UBM) and anterior segment optical coherence tomography (OCT) can clearly identify these cystic lesions. [2, 3]

**Table 1:** Classification of iris cysts

I.	Primary cyst		
	1. Iris pigment epithelium cyst	i. Pupillary	
		ii. Midzonal	
		iii. Peripheral	
		iv. Dislodged	
		v. Free-floating	1. Anterior chamber
			2. Vitreous chamber
	2. Iris stroma cyst	i. Congenital	
		ii. Acquired	
II.	Secondary cyst		
	1. Epithelial	i. Epithelial downgrowth cyst	1. Postsurgical
			2. Posttraumatic
		ii. Pearl cyst	
		iii. Drug-induced cyst	
	2. Cyst secondary to intraocular tumors	i. Medulloepithelioma	
		ii. Uveal melanoma	
		iii. Uveal nevus	
	3. Parasitic cyst		

Classification from Shields JA. Primary cysts of the iris. *Trans Am Ophthalmol Soc.* 1981;79:771–809

We report a rare case of bilateral IPE cysts in a male toddler who was subsequently diagnosed with B-cell acute lymphoblastic leukemia (B-cell ALL). Notably, the IPE cysts reduced in size and number after chemotherapy was initiated for the treatment of acute lymphoblastic leukemia (ALL).

## Case presentation

A 3-year-old male toddler initially presented to the emergency department with symptoms of anemia. He had a previous hospitalization for similar complaints but had subsequently defaulted on pediatric follow-up. On examination, he appeared tachypnoeic and pale. Multiple lymph nodes were palpable across his body, and hepatosplenomegaly was noted. There was no evidence of neurological or testicular involvement. He was admitted to the intensive care unit (ICU) for further monitoring. During his ICU stay, the pediatric team incidentally noted cystic lesions at the margins of both pupils, prompting referral to the ophthalmology team.

**Fig. 1:**
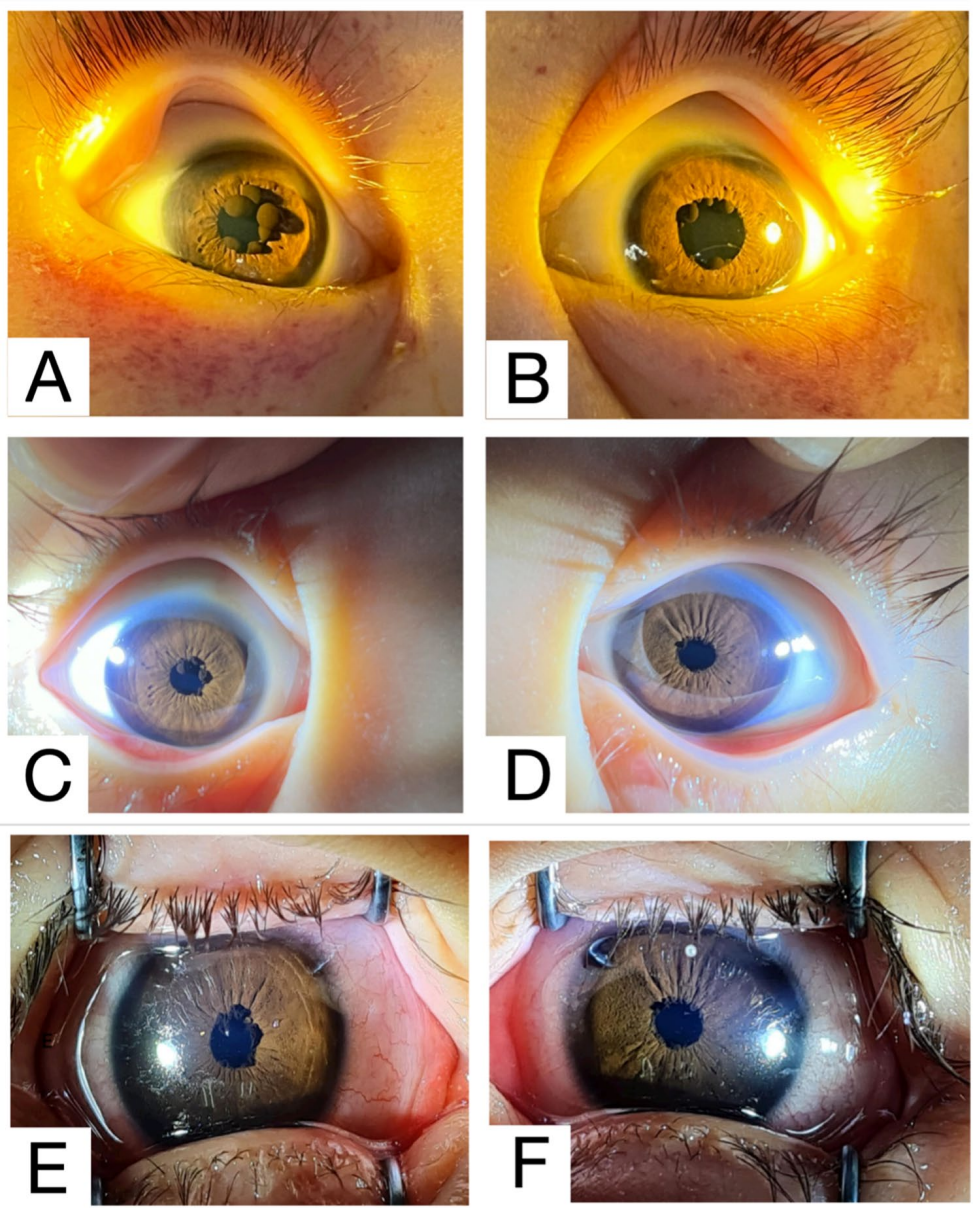
Both eyes anterior segment photography **(A)** Right eye showing IPE cysts before initiation of chemotherapy, with petechial rashes around the eyelids; **(B)** Left eye showing IPE cysts before initiation of chemotherapy, also with petechial rashes around the eyelids; **(C)** Right eye two months after starting chemotherapy, showing decreased size of the IPE cyst and resolution of petechial rashes; **(D)** Left eye two months after starting chemotherapy, showing significant reduction in size and number of IPE cysts and resolution of petechial rashes; **(E)** Right eye 18 months after chemotherapy initiation; **(F)** Left eye 18 months after chemotherapy initiation

On further inquiry, there was no family history of malignancy or similar ocular conditions. There was also no history of trauma, eye redness or eye procedure. The patient was not receiving any topical or systemic medications known to cause iris cysts. Neither the parents nor medical personnel had observed the presence of iris cysts since birth or during prior hospital admissions. Ophthalmic examination revealed bilateral IPE cysts (Fig. 1A and B). Although the images appeared consistent with pupillary cysts, their cystic nature could not be confirmed by ultrasound biomicroscopy owing to the unavailability of this facility. Fundus examination showed a dull macula, and tortuous vessels, with no retinal hemorrhages observed. No cysts were found in the fundus or vitreous during examination with indirect ophthalmoscopy. Intraocular pressure was within normal limits. Petechial rashes were present on both eyelids. B-scan ultrasonography confirmed a flat retina and no additional cysts.

In view of pancytopenia—white cell count 0.59 × 10³/µL, hemoglobin 1.7 g/dL, and platelet count 4 × 10³/µL—the pediatric team initiated a workup to rule out acute leukemia. Coagulation studies were within normal limits, with a prothrombin time of 32.1 s, activated partial thromboplastin time of 43.1 s, and an international normalized ratio (INR) of 2.62.

**Fig. 2:**
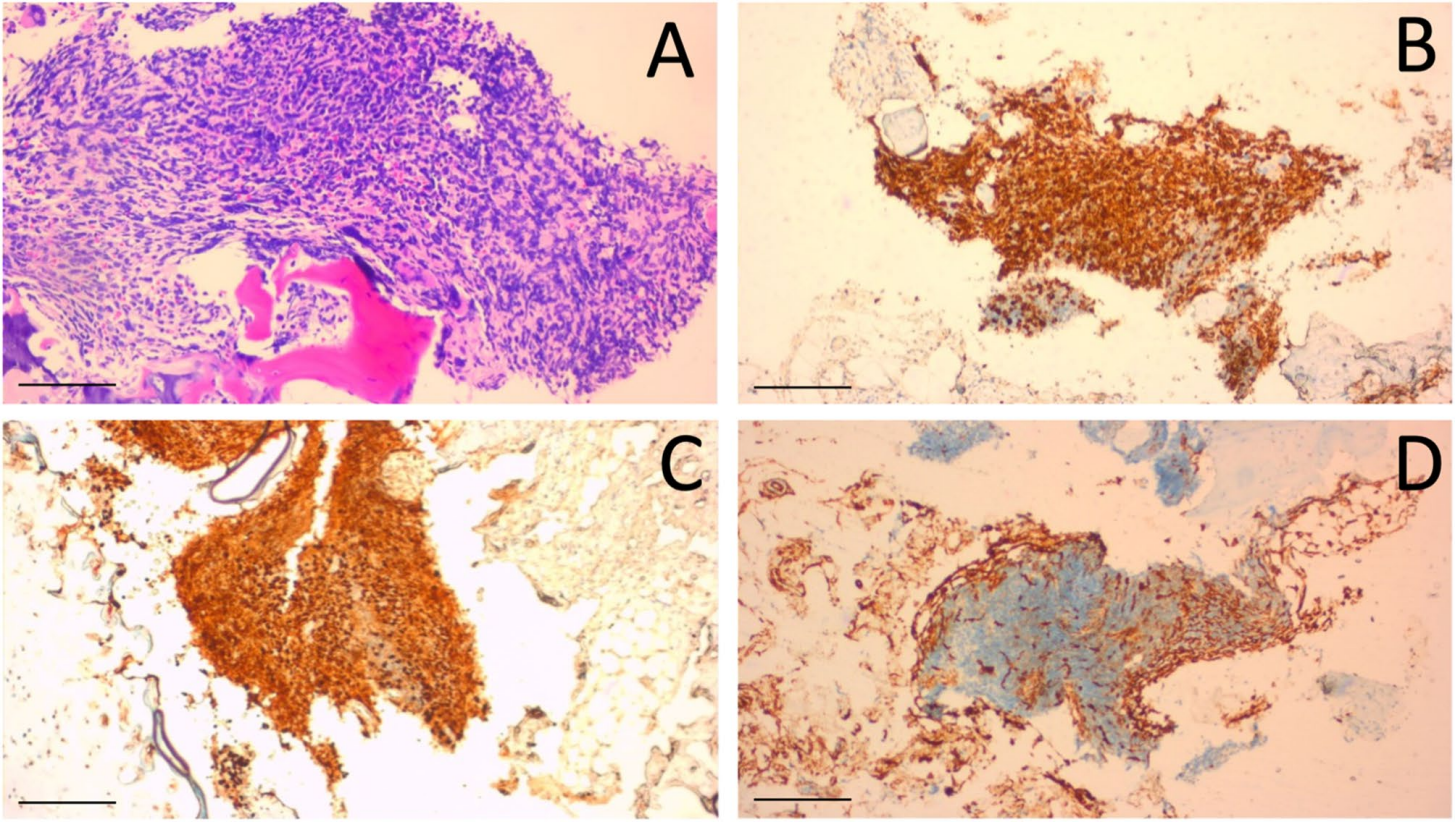
Diagnostic trephine biopsy **(A)** May-Grünwald-Giemsa (MGG) (x20); **(B)** CD79a positive (x10); **(C)** Terminal deoxynucleotidyl Transferase (TdT) positive (x10); **(D)** CD34 negative (x10); Trephine biopsy, these blasts are negative for CD3, CD117 & Myeloperoxidase (MPO); CD79a – pan marker for B cells; TdT – immature marker; CD34 – immature marker; CD3 - T cell marker; CD117 – tyrosine kinase growth factor receptor; MPO – Myeloid marker. Scale bars: 100 μm (A) and 200 μm (B-D)

**Fig. 3:**
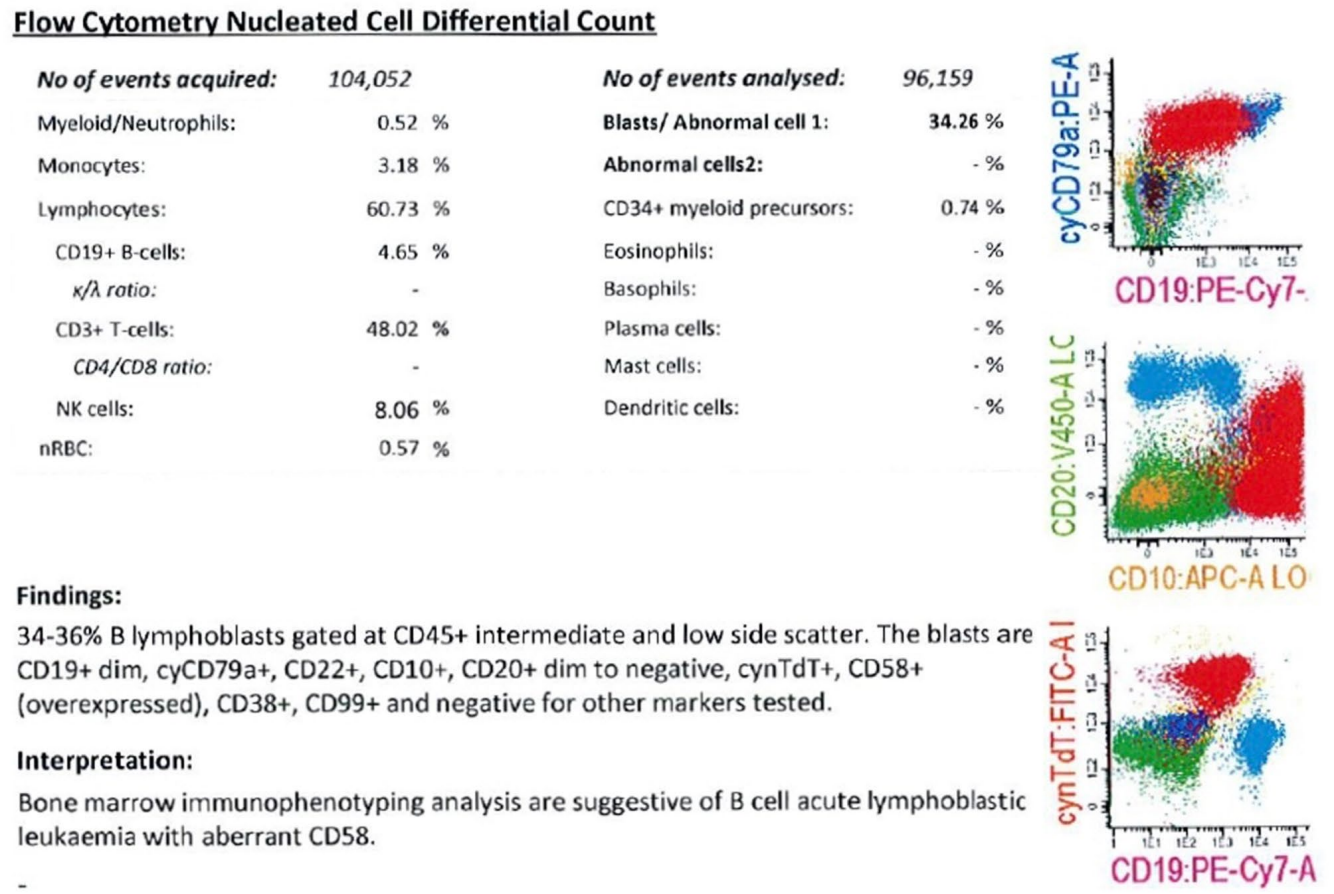
Bone marrow immunophenotyping analysis

Peripheral blood film (PBF) showed a leucoerythroblastic picture with no obvious blasts. Bone marrow aspirate and trephine (BMAT) biopsy report revealed 60–61% lymphoblasts. Diagnostic trephine biopsy (Fig. 2) May-Grünwald-Giemsa (MGG) showed infiltration by blasts and positive for CD79a and Terminal deoxynucleotidyl Transferase (TdT). However, the blasts were negative for CD34, CD3, CD117 and myeloperoxidase (MPO). Immunophenotyping (Fig. 3) confirmed B-cell ALL with aberrant CD58 expression. Cytogenetic analysis showed a hyperdiploid karyotype with 63–64 chromosomes and multiple extra copies including chromosomes X, Y, 3, 4, 5, 6, 7, 8, 10, 12, 14, 15, 17, 19, 21 and 22. This abnormal karyotype is consistent with hyperdiploidy, which is recognized favourable prognostic factor in pediatric ALL. No translocations or mutations were detected on molecular studies.

The patient was referred to the oncology team. He was commenced on the chemotherapy regimen Associazione Italiana Ematologia Oncologia Pediatrica (AIEOP) Berlin-Frankfurt-Münster (BFM) 2009 Protocol [5]. The IPE cysts began to shrink after two months of chemotherapy (Fig. 1C and D), with further size reduction noted after 18 months (Fig. 1E and F). He is currently in complete remission (CR) and minimal residual disease (MRD) negative.

## Discussion

Leukemias are malignant neoplasms resulting from the clonal proliferation of hematopoietic stem cells, characterized by diffuse replacement of the bone marrow by neoplastic cells. [6, 7] Leukemia can manifest in the eyes, and ocular involvement may be the initial presentation or the first sign of relapse of the systemic disease. [8]

Ophthalmic manifestations of leukemia are generally classified into two major categories:

(A) Primary or direct leukemic infiltration
(B) Secondary or indirect involvement [6]

Direct leukemic infiltration can present in three patterns: uveal infiltration, orbital infiltration, and neuro-ophthalmic signs of central nervous system leukemia, which include optic nerve infiltration, cranial nerve palsies, and papilloedema. [6]

Secondary ocular changes result from hematologic abnormalities associated with leukemia, such as anemia, thrombocytopenia, hyperviscosity, and immunosuppression. These changes can manifest as retinal or vitreous hemorrhages, infections, and vascular occlusions. [6–9]

Ocular involvement may be asymptomatic, particularly in pediatric cases, as young children often do not report visual symptoms. [6–10] Retinopathy was previously considered to have no prognostic significance in acute leukemia during the era before effective antileukemic therapy. [11] However, recent studies have shown that the presence of ocular involvement is associated with poorer prognosis in childhood acute leukemia. [6–11]

The literature regarding the association between IPE cysts and hematological malignancies is extremely limited. To our knowledge, only a single case has been reported in which IPE cysts were secondary to Hodgkin’s lymphoma, with the cysts decreasing in size following the initiation of chemotherapy. [12] Similarly, in our case, there was a notable reduction in the size and number of IPE cysts after the commencement of chemotherapy for ALL. The reduction in the size and number of IPE cysts may represent part of the natural history rather than a treatment effect of chemotherapy. This reduction could be attributed to cyst shrinkage and/or migration to the posterior segment. However, vitreous cysts were excluded by indirect ophthalmoscopy and ultrasonography during the follow-up visit.

The scarcity of reported cases may be attributed to the rarity of this phenomenon or to underreporting, possibly due to the difficulty of detecting IPE cysts during routine slit-lamp examinations. [12] Based on current findings, it is plausible that the IPE cysts were associated with the underlying hematological malignancy. To date, there have been no reported cases of IPE cysts in patients with leukemia. We hereby present the first documented case of IPE cysts in a patient with ALL.

IPE cysts can manifest in various locations: centrally at the pupillary margin (7%), midzonal (28%), peripherally (63%), or as free-floating cysts in the aqueous or vitreous, or lodged in the anterior chamber angle (2%) (Table 1). [2] Central IPE cysts at the pupillary margin are exclusively observed in males, while peripheral cysts are predominantly found in females (69%), with no gender preference noted for midzonal and dislodged cysts [4]. Central and peripheral IPE cysts typically present in younger patients, whereas midzonal and dislodged cysts tend to occur in older individuals [4]. 

In this patient, the IPE cysts were located bilaterally at the pupillary margin. Pupillary margin cysts are typically visible without pupil dilation and appear as round or collapsed brown lesions arising from the pupillary edge, often located superonasally [4]. Another variant of IPE cysts, known as iris flocculi, may be associated with systemic thoracic aortic aneurysm and has been linked to genetic mutations affecting the smooth muscle of both the iris and the aorta. [2]

The diagnosis of IPE cysts is typically made clinically during slit-lamp examination; however, clearer visualization can be achieved using UBM and anterior segment OCT. [2] Anterior segment OCT provides high-resolution imaging of the anterior cyst wall, while UBM offers slightly lower resolution but allows visualization of both the anterior and posterior cyst walls.².

As most IPE cysts follow a benign clinical course, treatment is rarely required unless the cysts obstruct the visual axis or block the anterior chamber angle [4]. Symptomatic cysts, however, may warrant intervention. [13]

It remains uncertain whether the IPE cysts in this patient were present since birth and simply went unnoticed by family members, as the child was too young to report any visual symptoms. However, the observed changes in the size and number of the IPE cysts during the course of treatment suggest a dynamic process. The reduction of the cysts following chemotherapy supports a possible association between the IPE cysts and the underlying leukemia.

## Conclusion

We report a rare case of IPE cysts in a patient with ALL, in which the cysts significantly reduced in size following the initiation of chemotherapy. This unusual finding highlights a potential association between IPE cysts and hematological malignancies, emphasizing the need for further research to elucidate their underlying pathogenesis and treatment implications. Moreover, this case underscores the importance of incorporating routine ophthalmic examinations into the evaluation of leukemia patients, given the high prevalence of asymptomatic ocular lesions in this population.

## Supplementary Information

Supplementary Material 1.

## Data Availability

No datasets were generated or analysed during the current study.

## Abbreviations

IPE cysts: Iris Pigment Epithelial cysts
ALL: Acute lymphoblastic leukemia
BE: Both Eyes
ICU: Intensive Care Unit
UBM: Ultrasound Biomicroscopy
OCT: Optical Coherence Tomography
INR: International Normalized Ratio
BMAT: Bone marrow aspiration and trephine biopsy
AEIOP: Associazone Italiana Ematologia Oncologia Pediatrica
BFM: Berlin -Frankfurt-Munster
CR: Complete Response
MRD: Minimal Residual Disease
