# Supplementary material for: Iris pigment epithelial cysts in acute lymphoblastic leukemia-a case report
Source: BMC Ophthalmol. 2025 Oct 17;25:582. doi: 10.1186/s12886-025-04419-8 (PMC12535150; doi:10.1186/s12886-025-04419-8)
Supplement: Supplementary file 1 — Supplementary Material 1. [file 12886_2025_4419_MOESM1_ESM.pdf]

CARE case report guidelines

CARE Checklist of information to include when writing a case report

(cc) BY-NC-ND

✓

Topic	Item	Checklist item description	Reported on Line
Title	1	The diagnosis or intervention of primary focus followed by the words "case report"	page 1, line 2
Key Words	2	2 to 5 key words that identify diagnoses or interventions in this case report, including "case report"	page 3, line 41-42
Abstract (no references)	3a	Introduction: What is unique about this case and what does it add to the scientific literature?	page 1, line 16-18
	3b	Main symptoms and/or important clinical findings	page 2, line 21-23
	3c	The main diagnoses, therapeutic interventions, and outcomes	page 2, line 31-33
	3d	Conclusion—What is the main "take-away" lesson(s) from this case?	page 2, line 36-38
Introduction	4	One or two paragraphs summarizing why this case is unique (**may include references**)	page 3, line 52-53
Patient Information	5a	De-identified patient specific information.	page 4, line 57
	5b	Primary concerns and symptoms of the patient.	page 4, line 57
	5c	Medical, family, and psycho-social history including relevant genetic information	page 5, line 64
	5d	Relevant past interventions with outcomes	page 5, line 75-78
Clinical Findings	6	Describe significant physical examination (PE) and important clinical findings.	page 4, line 59-62
Timeline	7	Historical and current information from this episode of care organized as a timeline	page 4, line 58
Diagnostic Assessment	8a	Diagnostic testing (such as PE, laboratory testing, imaging, surveys).	page 5, line 73-75
	8b	Diagnostic challenges (such as access to testing, financial, or cultural)	N/A
	8c	Diagnosis (including other diagnoses considered)	page 5, line 74-75
	8d	Prognosis (such as staging in oncology) where applicable	page 5, line 77-78
Therapeutic Intervention	9a	Types of therapeutic intervention (such as pharmacologic, surgical, preventive, self-care)	page 5, line 76-77
	9b	Administration of therapeutic intervention (such as dosage, strength, duration)	page 5, line 76-77
	9c	Changes in therapeutic intervention (with rationale)	page 5, line 77-78
Follow-up and Outcomes	10a	Clinician and patient-assessed outcomes (if available)	N/A
	10b	Important follow-up diagnostic and other test results	page 5, line 68-71
	10c	Intervention adherence and tolerability (How was this assessed?)	page 5, line 76-77
	10d	Adverse and unanticipated events	N/A
Discussion	11a	A scientific discussion of the strengths AND limitations associated with this case report	page 9, line 111
	11b	Discussion of the relevant medical literature **with references**.	page 9, line 112-114
	11c	The scientific rationale for any conclusions (including assessment of possible causes)	page 10, line 141-142
	11d	The primary "take-away" lessons of this case report (without references) in a one paragraph conclusion	page 11, line 144-149
Patient Perspective	12	The patient should share their perspective in one to two paragraphs on the treatment(s) they received	N/A
Informed Consent	13	Did the patient give informed consent? Please provide if requested	Yes ☑ No ☐
